# Relationship between *CYP2D6* genotype, activity score and phenotype in a pediatric Thai population treated with risperidone

**DOI:** 10.1038/s41598-021-83570-w

**Published:** 2021-02-18

**Authors:** Yaowaluck Hongkaew, Andrea Gaedigk, Bob Wilffert, Nattawat Ngamsamut, Wiranpat Kittitharaphan, Penkhae Limsila, Chonlaphat Sukasem

**Affiliations:** 1 Division of Pharmacogenomics and Personalized Medicine, Department of Pathology, Faculty of Medicine Ramathibodi Hospital, Mahidol University, Bangkok, 10400 Thailand; 2 Laboratory for Pharmacogenomics, Somdech Phra Debaratana Medical Center (SDMC), Ramathibodi Hospital, Bangkok, Thailand; 3 Advanced Research and Development Laboratory, Bumrungrad International Hospital, Bangkok, Thailand; 4 Division of Clinical Pharmacology, Toxicology and Therapeutic Innovation, Children’s Mercy Kansas City, Kansas City, MO USA; 5 School of Medicine, University of Missouri-Kansas City, Kansas City, MO USA; 6 Unit of PharmacoTherapy, -Epidemiology and -Economics, Groningen Research Institute of Pharmacy, University of Groningen, Groningen, The Netherlands; 7 Department of Clinical Pharmacy and Pharmacology, University of Groningen, University Medical Center Groningen, Groningen, The Netherlands; 8 Yuwaprasart Waithayopathum Child and Adolescent Psychiatric Hospital, Department of Mental Health Services, Ministry of Public Health, Samut Prakan, Thailand

**Keywords:** Genetic association study, Genetic markers, Paediatrics, Therapeutics

## Abstract

Recently, the Clinical Pharmacogenetics Implementation Consortium (CPIC) have revised recommendations for the translation of *CYP2D6* genotype to phenotype. Changes affect phenotype grouping, as well as the value used to calculate activity score for the CYP2D6*10 allele to better reflect the substantially decreased activity of this allele which is the most frequent allele found in Asian populations. This study aimed to evaluate whether the lower value for CYP2D6*10 as recommended, and the revised phenotype groupings improve the relationship between *CYP2D6* genotype and risperidone measures. One hundred and ninety-nine children and adolescents with autism treated with a risperidone-based regimen for at least four weeks were included. *CYP2D6* genotype was determined using the Luminex xTAG CYP2D6 Kit assay and translated into phenotype using different translation methods. Plasma concentrations of risperidone and 9-hydroxyrisperidone were measured using LC/MS/MS. Plasma levels of risperidone, risperidone concentration/dose ratio, and risperidone/9-hydroxyrisperidone ratio in patients with an activity score < 1 were significantly higher than those ≥ 1 (*P* value < 0.001 for all three parameters). Plasma risperidone levels and risperidone concentration/dose ratios were significantly higher in intermediate metabolizers (defined as AS = 0.25–0.75) than normal metabolizer (defined as AS = 1–2) patients (1.44 vs. 0.23 ng/ml, *P* < 0.001 and 1.63 vs. 0.29 ng/ml/ng, *P* < 0.001, respectively) as well as risperidone/9-hydroxyrisperidone ratio (0.20 vs. 0.04, *P* < 0.001). This is the first study in an Asian population utilizing the revised CPIC-recommended method for translating the *CYP2D6* genotype to phenotype. In addition to validating that *CYP2D6* genetic variation significantly impacts risperidone metabolism, we demonstrated that revised value for the CYP2D6*10 was superior for genotype to phenotype translation. However, at least for risperidone, subjects with an activity score of 1 presented as phenotypic normal, and not intermediate metabolizers, suggesting that phenotype classification is substrate dependent.

## Introduction

Cytochrome P450 (P450) 2D6 is a major drug-metabolizing enzyme expressed in the liver^1^. CYP2D6 catalyzes the hepatic metabolism of a large number of clinically important medications, including codeine, amitriptyline, fluvoxamine, risperidone, fluoxetine, aripiprazole, paroxetine, and dextromethorphan^2,3^. The *CYP2D6* gene is highly polymorphic. To date, over 130 allelic variants have been designated by the Pharmacogene Variation Consortium (PharmVar)^4,5^.

*CYP2D6* allele frequencies vary substantially among different ethnic and ancestral populations^6–9^. The decreased function CYP2D6*10 allele (100C > T, P34S) is the most common allele in East Asian populations, including Thai, Chinese, Taiwanese, Korean, Vietnamese, and Filipino^10–16^. This allele is also observed in other populations, including Europeans, Africans, and their descendants, its frequency, however, considerably lower^8^. Conversely, the nonfunctional CYP2D6*4 allele is more frequent in European populations but is rarely observed in Asian populations^8^.

*CYP2D6* genetic variation leads to a wide range of metabolic capacity ranging from no to increased activity. Based on their genotype, individuals are grouped into four phenotype groups, i.e., poor metabolizers (PMs), intermediate metabolizers (IMs), normal metabolizers (NMs), and ultrarapid metabolizers (UMs)^17^. The activity score system (AS) has been broadly accepted to translate the *CYP2D6* genotype into phenotype and the Clinical Pharmacogenetics Implementation Consortium (CPIC) and the Dutch Pharmacogenetics Working Group (DPWG) for their respective guidelines^18,19^. Briefly, each allele is assigned a value of 0, 0.5 or 1 reflecting no function, decreased or normal function, and the sum of the values provides the AS of a genotype. The previous CPIC translation method classified AS = 0 as PM, AS = 0.5 as IM, AS = 1 to 2 as NM, and > 2 as UM. In an effort to harmonize genotype to phenotype translation, a CPIC-led working group has recently published a revised method and recommends using this new method to translate genotype to phenotype^19^. One major change was downgrading the value used for activity score calculation of the decreased function CYP2D6*10 allele from 0.5 to 0.25 to more accurately reflect the dramatically decreased function of this allele. Furthermore, an AS of 1 is no longer categorized as NM, but as IM. While the new system has recently been applied to an in vitro study comprising mostly Caucasian liver tissue samples^20^, there are no investigations to date assessing the performance of the new method on any Asian populations with high frequencies of CYP2D6*10. There is also a paucity of information regarding the impact of substrate specificity on performance of the new translation method.

The use of a standardized method to infer phenotype from genotype is essential for test reporting and clinical implementation to prevent confusion and inconsistencies. We applied the new CPIC-recommended method to data obtained from risperidone (RIS)-treated Thai children and adolescents diagnosed with autism spectrum disorders (ASDs) and treated with RIS. Since the impact of *CYP2D6* genotype on plasma concentrations of RIS is well-established^21–25^, RIS is a well-suited drug to evaluate whether the new translation method is superior over the previous method.

The aims of this investigation were to demonstrate whether the revised value for CYP2D6*10 indeed improves the relationship between AS and RIS plasma drug levels and to assess whether phenotype groupings, as recommended by CPIC, are appropriate for RIS.

## Subjects and methods

### Patients

One hundred and ninety-nine participants with ASD, aged 3–18 years, and diagnosed according to the Diagnostic and Statistical Manual of Mental Disorders, Fifth Edition (DSM-V) criteria in the Yuwaprasart Waithayopathum Child Psychiatric Hospital, Samut Prakan, Thailand, were recruited during 2017–2018. All patients were treated with a RIS-based regimen for at least four weeks before blood sample collection. Socio-demographic data were collected by a questionnaire including gender, age at assessment, daily RIS dosage, duration of RIS treatment, and concomitant medication. Patients were excluded if they were receiving concomitant treatments that could potentially affect RIS metabolism. This study was approved by the Ethics Review Committee on Human Research of the Faculty of Medicine Ramathibodi Hospital, Mahidol University, Thailand (MURA2017/556) and conducted in accordance with the Declaration of Helsinki. The study protocol was clearly explained to all participants and/or their legal guardians, and informed consent was given before the study.

### Genotyping methods

Genomic DNA was extracted from EDTA blood with the MagNa Pure automated extraction system according to the manufacturer's instructions. A bead array platform genotyped *CYP2D6* based on allele-specific primer extension (ASPE) and hybridization to oligonucleotide bound microspheres^26^ using the Luminex xTAG CYP2D6 Kit v3 (Luminex Corporation, Austin, TX, USA) according to the manufacturer's instructions^27^. The assay interrogates 21 variants including 19 *CYP2D6* single nucleotide polymorphisms (SNPs): − 1584C > G, 31G > A, 100C > T, 124G > A, 137_138insT, 882G > C, 1022C > T, 1660G > A, 1662G > C, 1708delT, 1759G > T, 1847G > A, 2550delA, 2616delAAG, 2851C > T, 2936A > C, 2989G > A, 3184G > A, and 4181G > C, as well as gene deletion and duplication)^25^. The allelic variants called by this array are CYP2D6*1 (assigned in the absence of variants; default assignment), *2, *35 (normal function), *9, *10, *17, *29 and *41 (decreased function), and *3, * 4, *5, *6, *7, *8, *11 and *15 (no function), as well as the presence of duplications. Patients who were carriers of a *CYP2D6* duplication were excluded, because this array did not further characterize gene duplications (i.e. copy number or which allele is affected by the duplication). For instance, a duplication observed in an individual genotyped as CYP2D6*1/*10 could result in e.g. a CYP2D6*1xN/*10, CYP2D6*1/*10xN or a *1/*36 + *10 genotype call.

To calculate the AS, values were assigned to the alleles identified in the study cohort as follows: no function alleles (*4, *5) = 0; the decreased function allele *10 = 0.25; other decreased function alleles (*14, *41) = 0.5, and normal function alleles (*1, *2, *35) = 1. The AS of each diplotype is the sum of the assigned value to each allele. Individuals with an AS of 0 were categorized as PMs, those with an AS of 0.25, 0.5 or 0.75 were categorized as IMs, and those with an AS of 1.25, 1.5, 1.75, or 2 were grouped as NMs. To compare translation methods, those with an AS of 1 were either categorized as IM (new CPIC method), or NM (previous CPIC method).

### Analytical drug assay/plasma concentrations

Trough plasma concentration of RIS and its 9-OH-RIS metabolite were quantified, between 8:00 and 10:00 AM, approximately 12 h after the bedtime dose, using a validated, previously published high-performance liquid chromatography procedure^28^. Briefly, we used an Agilent 1260 HPLC system (Agilent Technologies, CA, USA), which was connected to an AB Sciex API 3200 (Applied Biosystems, Foster City, CA, USA) instrument. Chromatographic separation was achieved on the C18 column (4.6 cm × 50 mm; 1.8 mm particle size). Integration of peak areas and determination of the concentrations was performed with the Analyst 1.5.2 software (Applied Biosystems, CA, USA). Quadratic regression with 1/ × weighted concentrations was used. The mean inter- and intra-assay accuracy for both RIS and 9-OH-RIS was set within ± 15.0% Relative Error of nominal, and precision < 15.0% Relative Standard Deviation.

### Statistical analysis

Descriptive statistics were used to describe the clinical characteristics of the subjects. Data were expressed as mean (standard deviation, SD) or median (interquartile range, IQR) in normal or non-normal distribution data, respectively. The nonparametric Kruskal–Wallis (comparisons more than two groups) and Mann–Whitney U tests (comparisons between two groups) were used to assess the association between plasma drug levels and the studied genotypes or predicted phenotypes at each time point. Statistical analyses were carried out using SPSS v24 (SPSS Inc., Chicago, IL, USA) for Windows. Statistical significance is reported as *P* < 0.05 for a two-tailed distribution.

## Results

### Demographic and clinical characteristics

Our sample consisted of 199 children and adolescents with a mean age of 9.25 (SD; 3.93) years who had been diagnosed with autism spectrum disorders. Demographic data are presented in Table 1. Participants were treated with a RIS-based regimen. One hundred and eighteen patients (59.3%) received RIS monotherapy. The medications that were concomitantly prescribed to patients were methylphenidate, sodium valproic acid, benzhexol, topiramate, cetirizine, clonazepam, hypodine, phenytoin, and phenobarbital. There were no significant differences for RIS or 9-OH-RIS between children and adolescents. Most of which were male (174; 87.44%). There were also no significant differences for RIS or 9-OH-RIS between males and females nor those receiving monotherapy and polytherapy.

**Table 1:** Patient demographics (n = 199).

Clinical information	Value
Age (years); mean ± SD	9.25 ± 3.93
Male to female (M:F) ratio	7:1
Daily risperidone dosage (mg/day); median (range), ng/ml	0.75 (0.10–5.00)
Risperidone treatment duration (months); median (IQR), ng/ml	43.47 (16.40–76.60)
Risperidone monotherapy, n (%)	118 (59.30)
**Plasma drug levels, median (IQR), ng/ml**	
RIS level	0.59 (0.06–1.61)
9-OH-RIS level	5.78 (3.38–11.50)
Active moiety level	7.06 (4.26–12.89)
Ratio of risperidone/9-OH-RIS	0.08 (0.02–0.24)
**Plasma concentration-to-dose (C/D) ratios, median (IQR), ng/ml/mg**	
C/D of RIS	0.71 (0.17–2.25)
C/D of 9-OH-RIS	8.45 (5.34–12.65)
C/D of the active moiety	9.60 (6.20–15.76)

*RIS* risperidone, *9-OH-RIS* 9-hydroxyrisperidone, Active moiety, the sum of risperidone plus 9-OH-RIS, *C/D* dose-corrected concentration, *SD* standard deviation, *IQR* interquartile range.

### Distribution of the *CYP2D6* alleles and genotypes

The CYP2D6*10 decreased function allele was the most common allele identified among the 199 subjects at 51.8%. The frequencies of the normal function alleles CYP2D6*1 and CYP2D6*2 were 25.1% and 6.3%, respectively. Another decreased function allele, CYP2D6*41, was observed at 6.8%. CYP2D6*4 and CYP2D6*5, both nonfunctional alleles, were found at frequencies of 1.3% and 8.3%, respectively. We also observed two subjects with the rare CYP2D6*14 allele (0.50%) in this study cohort. *CYP2D6* allele frequencies are presented in Table 2. Of the 398 alleles, 125 were normal function (aggregate frequency of 31.4%) and were assigned a value of 1 to calculate the AS while 29 decreased function alleles (aggregate frequency of 7.3%) received a value of 0.5 and 38 no function alleles (aggregate frequency of 9.6%) received a value of 0.

**Table 2:** *CYP2D6* allele frequencies (n = 199).

Alleles	CPIC clinical function	Frequency (%)
*1	Normal function	100 (25.1%)
*2	Normal function	25 (6.3%)
*4	No function	5 (1.3%)
*5	No function	33 (8.3%)
*10	Decreased function	206 (51.8%)
*14	Decreased function	2 (0.5%)
*41	Decreased function	27 (6.8%)

Allele definitions are per PharmVar at https://www.pharmvar.org/gene/CYP2D6.

Genotype frequencies are summarized in Supplementary Table 1. Of the 20 *CYP2D6* genotypes identified, CYP2D6*1/*10 was the most frequent (29.6%), followed by CYP2D6*10/*10, CYP2D6*5/*10, and CYP2D6*10/*41 (26.1%, 7.5%, and 7.5%, respectively).

### Plasma levels and C/D of RIS, 9-OH-RIS, active moiety, and RIS/9-OH-RIS ratio in the different CYP2D6 AS groups

**Figure 1:**
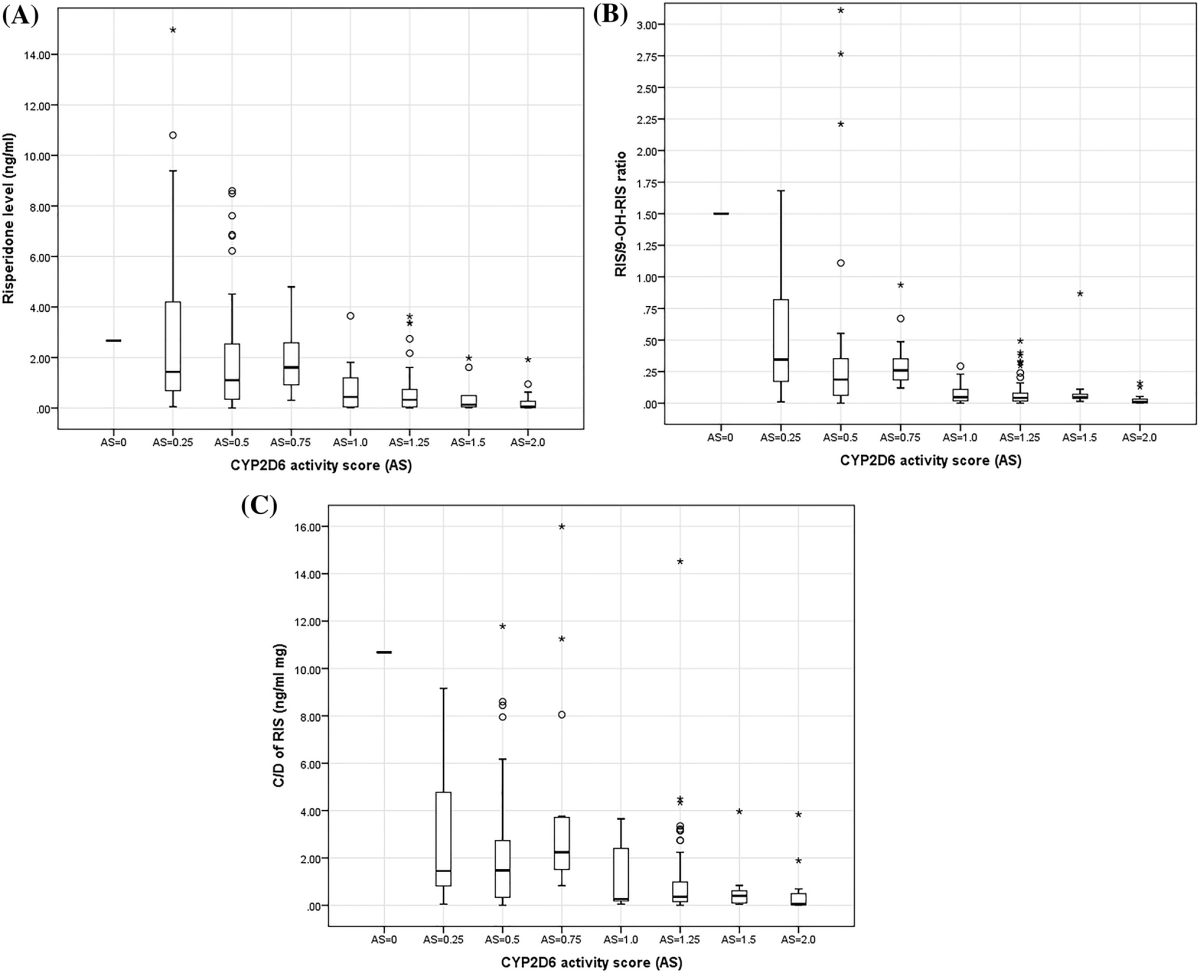
Plasma levels of RIS (A), RIS/9-OH-RIS ratio (B), and C/D of RIS (C) among activity score (AS) groups. AS was calculated using a value of 0.25 for the decreased function CYP2D6*10 allele.

The relationship between CYP2D6 AS, RIS plasma concentration, and the 9-OH-RIS metabolite was examined in 199 patients (Table 3). Patients were divided into eight groups (AS of 0, 0.25, 0.5, 0.75, 1, 1.25, 1.5, and 2). The most common AS was 1.25 (35.18%), comprising CYP2D6*1/*10 and CYP2D6*2/*10 genotypes. There were significant differences in RIS, the metabolic ratio RIS/9-OH-RIS, and C/D of RIS plasma concentrations between AS of 0.25, 0.5, 0.75, and 1, 1.25, 1.5, 2. There was a significant difference between patients when divided into two groups, one with AS < 1 and the other with AS ≥ 1. Plasma levels of RIS and RIS/9-OH-RIS ratio, and plasma C/D of RIS in patients with AS < 1 were significantly higher than those in patients with AS ≥ 1 (*P* value < 0.001 among three drug parameters) (Fig. 1A–C). When genotypes with an AS of 1 were categorized as IM, significance of RIS, RIS/9-OH-RIS ratio, and RIS C/D between AS of 1 and AS > 1 was considerably lower as reflected by a *P* value of 0.412, 0.519, and 0.314, compared to a *P* value of 0.005, 0.000, and 0.015 between AS of 1 and AS < 1. Based on these findings, individuals with an AS of 1 presented as NMs rather than IMs, while all others fit within their respective phenotype categories.

**Table 3:** Plasma levels and C/D of RIS, 9-OH-RIS, active moiety, and RIS/9-OH-RIS ratio among CYP2D6 activity scores groups (n = 199).

AS	n (%)	RIS (ng/ml)	9-OH-RIS (ng/ml)	Active moiety (ng/ml)	Ratio of RIS/9-OH-RIS	C/D of RIS (ng/ml/mg)	C/D of 9-OH-RIS (ng/ml/mg)	C/D of active moiety (ng/ml/mg)
AS = 0	1 (0.50)	2.67	1.78	4.45	1.50	10.68	7.12	17.80
AS = 0.25	17 (8.54)	1.43 (0.68–4.20)a	5.11 (3.86–13.56)	8.17 (4.53–22.95)	0.35 (0.17–0.82)a	1.45 (0.82–4.77)a	5.50 (3.86–8.00)d	9.01 (5.47–16.34)
AS = 0.5	55 (27.64)	1.10 (0.35–2.54)a	5.32 (3.11–11.40)	6.11 (4.54–11.96)	0.19 (0.06–0.35)a	1.48 (0.34–2.73)a	8.13 (5.42–11.93)	10.23 (6.32–14.39)
AS = 0.75	15 (7.54)	1.61 (0.92–2.58)a	5.13 (3.78–7.45)	7.25 (5.28–9.76)	0.26 (0.19–0.35)a	2.24 (1.52–3.71)a	10.15 (6.33–16.00)	11.68 (7.83–19.87)
AS = 1.0	17 (8.54)	0.44 (0.05–1.20)	7.56 (2.71–11.63)	7.56 (2.79–12.83)	0.05 (0.02–0.11)	0.27 (0.19–2.40)	10.90 (6.68–21.40)	11.79 (6.81–21.48)
AS = 1.25	70 (35.18)	0.33 (0.05–0.74)	7.27 (4.30–11.23)	7.60 (4.31–12.78)	0.04 (0.02–0.08)	0.36 (0.15–0.99)	9.18 (6.74–14.13)	9.72 (6.74–15.05)
AS = 1.5	9 (4.52)	0.13 (0.05–0.49)	2.28 (1.44–9.75)	4.19 (1.44–9.88)	0.05 (0.04–0.07)	0.40 (0.10–0.61)	6.74 (2.88–8.45)	8.38 (2.88–9.06)
AS = 2.0	15 (7.54)	0.05 (0.02–0.27)	8.36 (6.36–12.87)	8.36 (6.71–13.61)	0.01 (0.00–0.03)	0.05 (0.03–0.49)	7.95 (5.28–19.45)	8.08 (5.33–21.13)

^a^Statistically significant result (*P* < 0.05) from AS = 1.0, 1.25, 1.5, and 2.0.

Values expressed as median (interquartile range).

*AS* activity score (assigned per revised CPIC recommendations), *C/D* dose-corrected concentration, *RIS* Risperidone, *9-OH-RIS* 9-hydroxy-risperidone, Active moiety, the sum of risperidone plus 9-OH-RIS.

### Association between plasma RIS parameters and predicted phenotypes

Based on the above findings, patients with an AS of 0, AS of 0.25–0.75, and AS of 1–2 presented as, and were thus classified, as PM, IM, and NM, respectively. Fifty-six percentages of patients (n = 111) were NMs, followed by IMs (n = 87, 43.7%). There was only one patient with a predicted PM phenotype of 0.5%.

There were statistically significant differences for the plasma RIS concentration (*P* < 0.001) and RIS/9-OH-RIS ratio (*P* < 0.001) when subjects were categorized as described above (Table 4 and Fig. 1). The plasma concentration of RIS among IMs (AS = 0.25–0.75, 1.44 ng/ml) was significantly higher compared to that among NMs (AS = 1–2, 0.25 ng/ml, *P* < 0.001) and lower when compared to that found in the PM individual (2.67 ng/ml). The RIS/9-OH-RIS ratio in IM subjects was statistically significantly higher than the ratio observed in the NMs (AS = 1–2, 0.20 vs. 0.04, *P* < 0.001). These patients also had a significantly higher C/D of RIS than NMs (1.63 vs. 0.29 ng/ml/mg, *P* < 0.001).

**Table 4:** Plasma levels and C/D ratios of RIS, 9-OH-RIS, active moiety, and RIS/9-OH-RIS ratio among CYP2D6 phenotype groups (n = 199).

CYP2D6 predicted phenotype	n (%)	RIS (ng/ml)	9-OH-RIS (ng/ml)	Active moiety (ng/ml)	Ratio of RIS/9-OH-RIS	C/D of RIS (ng/ml/mg)	C/D of 9-OH-RIS (ng/ml/mg)	C/D of active moiety (ng/ml/mg)
PM	1 (0.5)	2.67	1.78	4.45	1.50	10.68	7.12	17.80
IM	87 (43.7)	1.44 (0.65–2.95)	5.22 (3.57–11.40)	6.50 (4.54–14.40)	0.20 (0.13–0.37)	1.63 (0.83–3.66)	7.74 (5.24–11.40)	9.85 (6.20–15.34)
NM	111 (55.8)	0.25 (0.05–0.74)	7.33 (3.68–11.67)	7.88 (4.01–12.89)	0.04 (0.01–0.08)	0.29 (0.09–0.93)	9.03 (6.28–14.13)	9.50 (6.43–16.04)
*P* value^a^		< 0.001^a^	0.185	0.836	< 0.001^a^	< 0.001^a^	0.105	0.879

^a^Statistically significant (*P* < 0.05) between CYP2D6 IM and NM.

CYP2D6 PM, AS = 0; IM, AS = 0.25, 0.5, and 0.75; NM, AS = 1.0, 1.25, 1.5 and 2.0.

*PM* poor metabolizer, *IM* intermediate metabolizer, *NM* normal metabolizer, *C/D* dose-corrected concentration, *RIS* Risperidone, *9-OH-RIS* 9-hydroxy-risperidone, Active moiety, the sum of RIS plus 9-OH-RIS.

## Discussion

To the best of our knowledge, this is the first study applying the revised CPIC recommendations for the translation of *CYP2D6* genotype to phenotype in an Asian population. This new method is anticipated to have a considerable impact on Asians compared to other populations due to the high frequency of the CYP2D6*10 allele. This allele conveys a considerable decrease in function and thus was downgraded, i.e., now receives a lower value for AS calculation, to improve the accuracy of phenotype prediction. The CPIC recommendations are drug-agnostic, i.e., the phenotype does not take substrate-specificity into account. Thus, in addition to evaluating whether the revised value for CYP2D6*10 improves the relationship between RIS, RIS/9-OH-RIS ratio, and C/D of RIS, we also assessed whether phenotype groupings, as recommended by CPIC, are appropriate for RIS.

**Figure 2:**
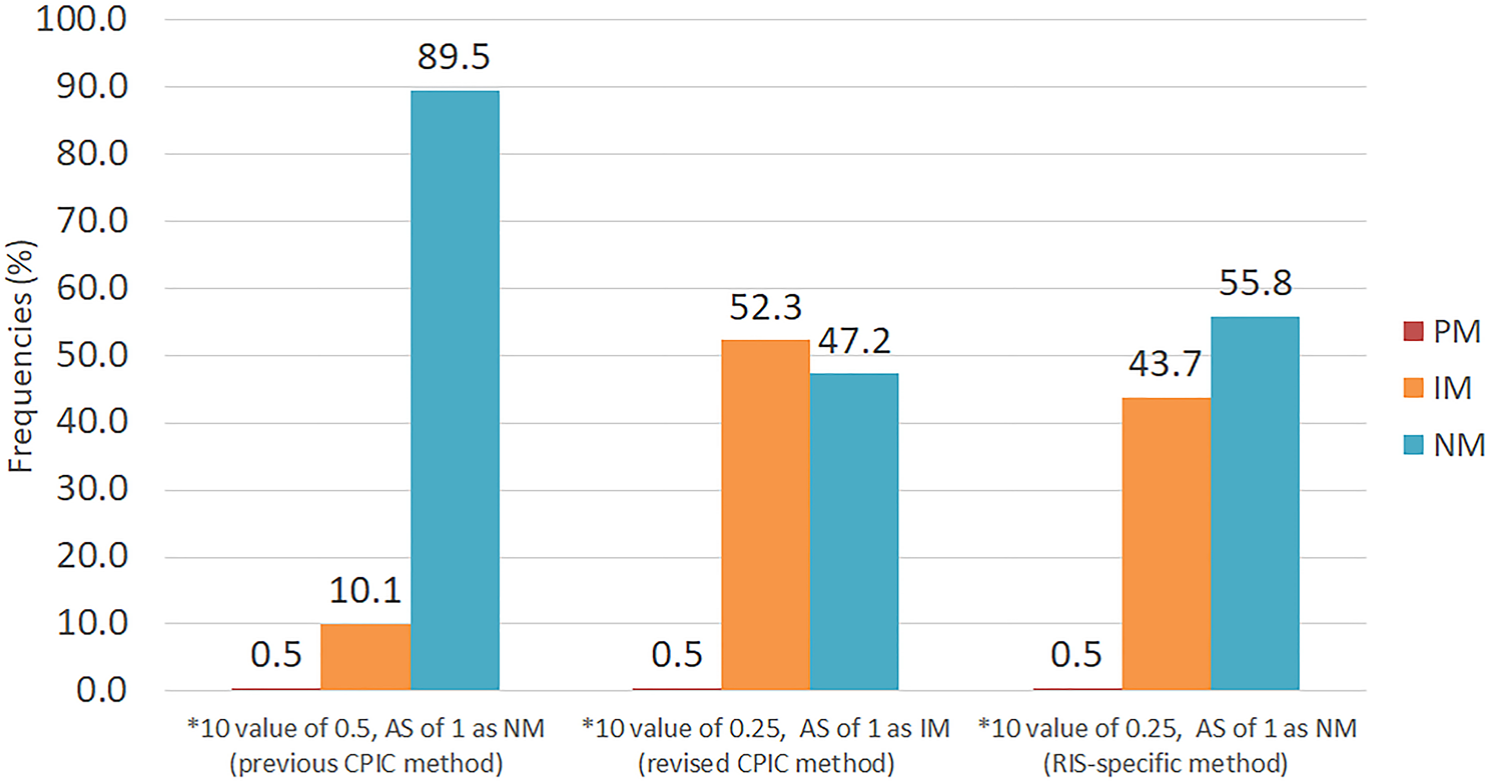
Frequencies of each predicted phenotypes using different genotype to phenotype translation methods. The previous CPIC method assigned CYP2D6*10 a value of 0.5 and classified subjects with an AS of 1 as NMs while the revised CPIC method assigns a value of 0.25 to the CYP2D6*10 allele and classifies subjects with an AS of 1 as IMs. This study used a value of 0.25 for CYP2D6*10 and classified subjects with an AS of 1 as NMs to predict phenotype for RIS. PM, poor metabolizer; IM, intermediate metabolizer; NM, normal metabolizer.

Owing to the revised AS definition, a notable number of subjects would be reclassified as IMs (Fig. 2). Specifically, 17 subjects with an AS of 1 which were grouped as NM under the old method would be grouped as IMs under the new method. Their observed phenotype, however, identified them as NMs suggesting that the recommended classification system does not improve phenotype prediction for RIS. In contrast, using the lower value of 0.25 for CYP2D6*10 AS calculation did improve the relationship between AS and RIS, RIS/9-OH-RIS ratio, and C/D of RIS. Similar findings were observed by Brown et al. who showed that systemic exposure of atomoxetine (AUC0-∞) of AS of 1 was not significantly different from that observed for subjects with an AS of 1.5 or 2^29^. In addition, Frederiksen et al^30^, demonstrated allele-specific metabolism of vortioxetine suggesting substantial differences among decreased function allele. Taken together, these findings raise awareness of the limitations and pitfalls of drug-agnostic genotype to phenotype translation methods. This is further substantiated by the plasma concentrations of RIS and RIS/9-OH-RIS ratios being significantly higher in AS of 0.25–0.75 than AS of 1–2 arguing that the former should be classified as IMs and the latter as NMs. Therefore, to predict CYP2D6 phenotype for RIS treatment, genotype should be translated into phenotype as shown in Table 5.

**Table 5:** Comparisons of CYP2D6 predicted phenotypes translation according to CYP2D6 activity score.

CYP2D6 predicted phenotype	Previous DPWG activity score definition	Previous CPIC activity score definition	Revised CPIC and DPWG activity score definition	This study (Thai autism cohort)
Ultrarapid metabolizer (UM)	> 2.5	> 2	> 2.25	N/A
Normal metabolizer (NM)	1.5–2.5	1–2	1.25–2.25	1–2
Intermediate metabolizer (IM)	0.5–1	0.5	0.25–1	0.25–0.75
Poor metabolizer (PM)	0	0	0	0

*N/A* not available.

Additionally, the *CYP2D6* genotype (or AS) had a substantial impact on the trough dose-corrected plasma concentration of RIS. In accordance with results we previously reported for a different cohort, there were statistically significant differences in the plasma concentration for RIS (*P* < 0.001) and the RIS/9-OH-RIS ratio (*P* < 0.001) among phenotype groups in Thai autism children^25,31^. Furthermore, PM patients had significantly higher RIS C/D than those genotyped as CYP2D6*1/*1^32^. The same pattern was also observed in another study^33^, i.e., the C/D ratio for RIS was significantly different in CYP2D6 PMs. The presence of the CYP2D6*10 allele was also associated with significantly higher levels of C/D of RIS levels at week 12 (*P* = 0.003) in North Indian patients with schizophrenia^34^. Moreover, plasma RIS/9-OH-RIS ratios were significantly higher in patients with an AS of 0.5 compared to those with an AS of 2 in an independent cohort of Thai subjects^24^. Taken together, the RIS/9-OH-RIS metabolic ratio is a biomarker for CYP2D6 activity, which may be useful to guide the treatment of patients in need of psychotropic drugs^35^.

There were no significant differences in 9-OH-RIS and total active moiety concentrations among the CYP2D6 predicted phenotype groups, as found in an earlier study^32^. Similarly, the total active moiety, sum of the plasma concentrations of RIS and 9-OH-RIS, corrected for the dose, did not significantly differ between individuals of different genotypes. These findings are consistent with a previous study in another Thai cohort of ASD patients^25,31^ that showed no significant differences in 9-OH-RIS and active moiety concentrations. This finding is consistent with a previous study using positron emission tomography scans of healthy volunteers after receiving a single oral dose of RIS showing that plasma concentrations of the sum of RIS and 9-OH-RIS partly overlapped between the NMs and PMs^36^. Therefore, the plasma concentrations of the 9-OH-RIS and total active moiety are independent of the CYP2D6-related metabolism. It has been suggested that the efflux transporter ABCB1, as well as CYP3A5 can contribute to the steady-state plasma concentration of RIS, 9-OH-RIS, and active moiety^37,38^*.*

As mentioned above, the CPIC-recommended drug-agnostic method to predict phenotype may not accurately predict phenotype across all drugs and all allelic variants. Regardless of the imperfections and shortcomings of the method, using a standardized system, although imperfect, is preferable because it makes comparisons of results among studies easier. However, it also demonstrates the need to develop more sophisticated algorithms that take substrate specificity, among other patient-specific information, into account.

We acknowledge the following limitations of the Luminex platform. This test does not quantitatively determine copy number nor does it determine which allele is duplicated or identify any other structural variants. Furthermore, only the most common alleles are tested. We speculate that some subjects may have rare or novel alleles which may explain some of the outliers shown in Fig. 1. In conclusion, the new CPIC recommended genotype to phenotype translation method, developed to promote standardized phenotype classification has its limitations for RIS. Using AS, rather than phenotype may be more accurate for this drug, especially considering the broad range of CYP2D6 activity and substrate specify. The findings of our study provide valuable information to further the implementation of genotype-guided risperidone treatment.

## Supplementary Information

Supplementary Information 1.
