# Supplementary material for: Relationship between CYP2D6 genotype, activity score and phenotype in a pediatric Thai population treated with risperidone
Source: Sci Rep. 2021 Feb 18;11:4158. doi: 10.1038/s41598-021-83570-w (PMC7892547; doi:10.1038/s41598-021-83570-w)
Supplement: Supplementary file 1 — Supplementary Information 1. [file 41598_2021_83570_MOESM1_ESM.docx]

**Relationship between CYP2D6 genotype, activity score and phenotype in a pediatric Thai population treated with risperidone**

Running title: CYP2D6 phenotype prediction using risperidone

Yaowaluck Hongkaew^1,2,3^, Andrea Gaedigk^4^, Bob Wilffert^5,6^, Nattawat Ngamsamut^7^, Wiranpat Kittitharaphan^7^, Penkhae Limsila^7^, Chonlaphat Sukasem^1,2*^

**Supplementary Table 1 *CYP2D6* genotype frequencies (n=199), values assigned to each allele for Activity Score calculation (per revised CPIC recommendations), and the activity score of each genotype.**

| **Genotype** | **n (%)** | **value allele 1** | **value allele 2** | **Activity score** |
| --- | --- | --- | --- | --- |
| **1/*1* | 8 (4.0) | 1 | 1 | 2 |
| **1/*2* | 6 (3.0) | 1 | 1 | 2 |
| **1/*4* | 1 (0.5) | 1 | 0 | 1 |
| **1/*5* | 12 (6.0) | 1 | 0 | 1 |
| **1/*10* | 59 (29.6) | 1 | 0.25 | 1.25 |
| **1/*41* | 6 (3.0) | 1 | 0.5 | 1.5 |
| **2/*2* | 1 (0.5) | 1 | 1 | 2 |
| **2/*4* | 1 (0.5) | 1 | 0 | 1 |
| **2/*5* | 2 (1.0) | 1 | 0 | 1 |
| **2/*10* | 11 (5.5) | 1 | 0.25 | 1.25 |
| **2/*14* | 1 (0.5) | 1 | 0.5 | 1.5 |
| **2/*41* | 2 (1.0) | 1 | 0.5 | 1.5 |
| **4/*5* | 1 (0.5) | 0 | 0 | 0 |
| **4/*10* | 2 (1.0) | 0 | 0.25 | 0.25 |
| **5/*10* | 15 (7.5) | 0 | 0.25 | 0.25 |
| **5/*14* | 1 (0.5) | 0 | 0.5 | 0.5 |
| **5/*41* | 2 (1.0) | 0 | 0.5 | 0.5 |
| **10/*10* | 52 (26.1) | 0.25 | 0.25 | 0.5 |
| **10/*41* | 15 (7.5) | 0.25 | 0.5 | 0.75 |
| **41/*41* | 1 (0.5) | 0.5 | 0.5 | 1 |
